# Synthesis of Multifunctional Nanoparticles for the Combination of Photodynamic Therapy and Immunotherapy

**DOI:** 10.3390/ph14060508

**Published:** 2021-05-26

**Authors:** Mei-Hwa Lee, James L. Thomas, Jin-An Li, Jyun-Ren Chen, Tzong-Liu Wang, Hung-Yin Lin

**Affiliations:** 1 Department of Materials Science and Engineering, I-Shou University, Kaohsiung 84001, Taiwan; 2 Department of Physics and Astronomy, University of New Mexico, Albuquerque, NM 87131, USA; jthomas@unm.edu; 3 Department of Chemical and Materials Engineering, National University of Kaohsiung, Kaohsiung 81148, Taiwan; m1075612@mail.nuk.edu.tw (J.-A.L.); 4a140109@stust.edu.tw (J.-R.C.); tlwang@nuk.edu.tw (T.-L.W.)

**Keywords:** immunotherapy, photodynamic therapy, programmed death-ligand 1 protein (PD-L1), magnetic nanoparticles, peptide-imprinted polymer

## Abstract

Programmed death-ligand 1 protein (PD-L1) has been posited to have a major role in suppressing the immune system during pregnancy, tissue allografts, autoimmune disease and other diseases, such as hepatitis. Photodynamic therapy uses light and a photosensitizer to generate singlet oxygen, which causes cell death (phototoxicity). In this work, photosensitizers (such as merocyanine) were immobilized on the surface of magnetic nanoparticles. One peptide sequence from PD-L1 was used as the template and imprinted onto poly(ethylene-*co*-vinyl alcohol) to generate magnetic composite nanoparticles for the targeting of PD-L1 on tumor cells. These nanoparticles were characterized using dynamic light scattering, high-performance liquid chromatography, Brunauer-Emmett-Teller analysis and superconducting quantum interference magnetometry. Natural killer-92 cells were added to these composite nanoparticles, which were then incubated with human hepatoma (HepG2) cells and illuminated with visible light for various periods. The viability and apoptosis pathway of HepG2 were examined using a cell counting kit-8 and quantitative real-time polymerase chain reaction. Finally, treatment with composite nanoparticles and irradiation of light was performed using an animal xenograft model.

## 1. Introduction

Molecularly imprinted polymers (MIPs) have been synthesized for use as biomimetic antibodies for bioseparation [1,2], biosensing [3,4,5] or gene activation [6,7]. The targets chosen for imprinting have been small molecules [5], proteins [5,8], or even cells [9,10]. Templates used included entire molecules [3,11], or epitopes [5,12] of targets. Especially with proteins, epitope imprinting is often used owing to the high cost of proteins, or solubility issues [1,13]. The specific recognition capabilities of MIPs are comparable to that of natural antibodies, and their stability is generally much better [14].

The science of protein epitope selection [5] or rational MIP design is immature, but several groups have demonstrated notable successes. Shea’s group employed peptides that contain nine amino acids from the C-terminus of two proteins (melittin and green fluorescent protein (GFP)) to prepare MIP nanoparticles [15,16]. Zhang’s group also used the C-terminal peptide for the recognition of albumin [17,18]. Kunter’s group selected epitopes from the crystal database of proteins that could be digested with various proteinases [19], and then the epitopes on the outside of the proteins were chosen for the synthesis of MIPs (used as sensing elements on an extended-gate field-effect transistor [20,21].) Our earlier investigations have also demonstrated epitope-based recognition of a protein using peptide-imprinted polymers [5,13,22]. MIPs may also be employed as artificial receptors for the recognition of surface ligands of cells [23,24]. 

Immune checkpoint pathways, including the programmed death receptor-1/programmed death ligand-1 (PD-1/PD-L1) signaling pathway [25], are important in mediating self-tolerance and controlling self-damage. This pathway can sometimes be manipulated by cancer cells to evade immune surveillance [26]. PD-L1 binds to its receptor, PD-1 on activated T cells, B cells and myeloid cells, to modulate activation or inhibition [27]. Recently, cancer immunotherapies [28,29,30] that train or stimulate immunological systems to recognize, attack, and eradicate tumor cells with minimal damage to healthy cells have yielded promising clinical responses; those that involve nanoparticles [29,31] for chemotherapy, radiotherapy-, photothermal therapy, photodynamic therapy and hyperthermia are especially effective [31]. The development of strategies to block PD-L1-mediated immune inhibition [32] could further enhance the effectiveness of immunotherapy.

Photodynamic therapy (PDT) uses light and a photosensitizer to elicit cell death (phototoxicity) [33]. Singlet oxygen (^1^O_2_), as the major reactive oxygen species (ROS), is produced in PDT to treat cancer [34]; mechanisms of action include direct cytotoxic effects exerted on tumor cells, destruction of the tumor and peritumoral vasculature, and induction of local acute inflammatory reaction [35]. The commonly used photosensitizer merocyanine 540 (MC 540) [36] is a member of the family of benzoxazol merocyanine dyes that contain heterocyclic aromatic groups linked by a polymethine chain. It has recently been used in studies of up-converting nanoparticles (UCNP) [37,38]. 

Hepatocellular carcinoma (HCC) is a primary malignancy of the liver, occurring mostly in patients with underlying chronic liver disease and cirrhosis [39]. Liver cancer is one of the leading causes of cancer deaths globally, and an annual death toll of 700,000 has been recorded in recent years [40]. Hepatocellular carcinoma treatments include surgery, liver transplantation, the destruction of cancer cells using heat or cold, the delivery of chemotherapy, radiation therapy, targeted drug therapy, immunotherapy, and new liver cancer treatments currently in clinical trials [41,42]. A combination of treatment modalities, rather than a single treatment, may be most effective in slowing the progression of the disease in humans.

**Figure 1:**
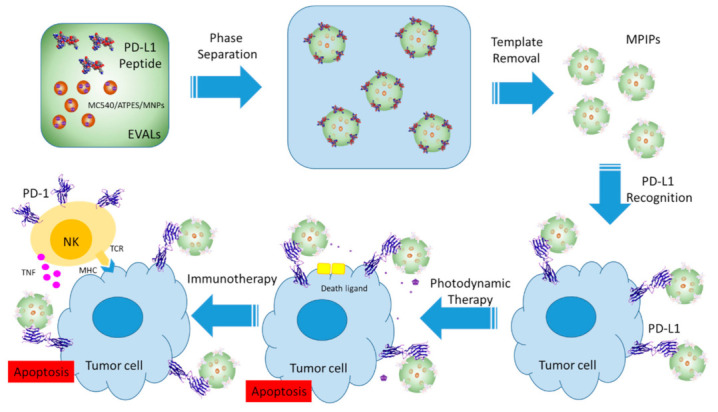
Preparation and administration of multifunctional magnetic PD-L1 peptide-imprinted composite nanoparticles.

In the present work, multifunctional MIP NPs were synthesized for cancer cell destruction. As shown in Figure 1, merocyanine (MC540) molecules were immobilized on the surface of magnetic nanoparticles (MNPs). These MC540/MNPs were then mixed with a peptide from PD-L1 and poly(ethylene-*co*-vinyl alcohol) solution to form multifunctional magnetic peptide-imprinted composite nanoparticles (MPIPs/MC540). These MPIPs were characterized for their size distribution, recognition capacity, specific surface area, and magnetization. Natural killer-92 (NK-92) cells were added to human hepatoma (HepG2) cells, which were then incubated with these composite nanoparticles and illuminated with visible light for varying durations. The viability and apoptosis pathway of HepG2 were investigated using cell counting kit-8 (CCK8) and quantitative real-time polymerase chain reaction (qRT-PCR). Finally, treatment with composite nanoparticles and irradiation of light was performed on an animal xenograft model.

## 2. Results and Discussion

**Figure 2:**
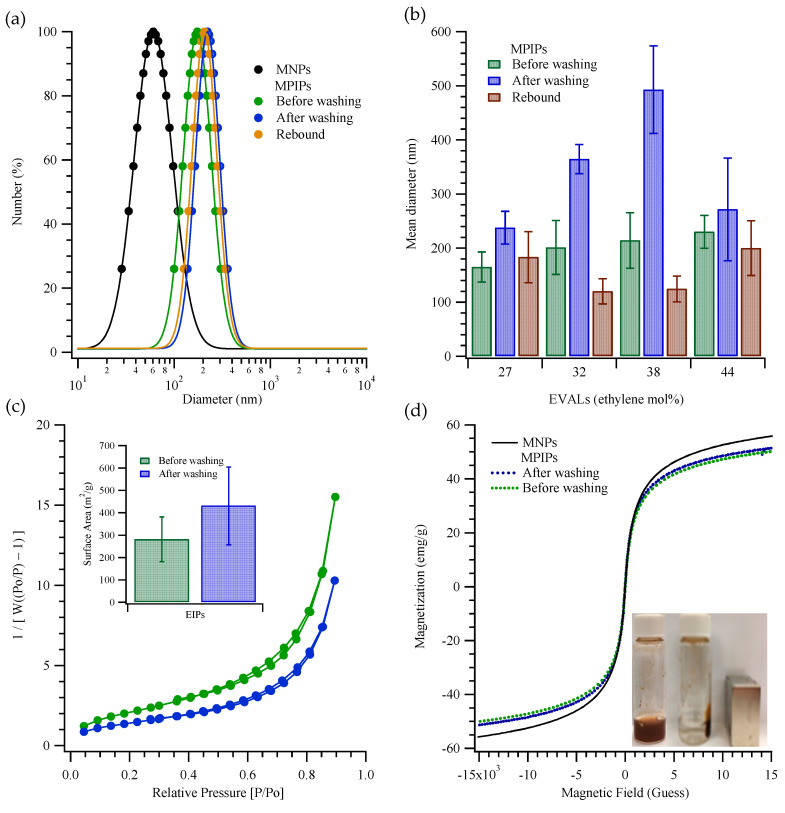
(**a**) Particles size distributions of MNPs (●); MPIPs before (●), after (●) template removal and rebound with template (●). (**b**) Mean diameters of MPIPs before, after template removal and rebound with template containing various ethylene mol% of EVAL. (**c**) Surface area of MPIPs before (●) and after (●) template removal measured by adsorption and desorption of nitrogen. (**d**) Magnetization of MNPs and MPIPs before and after template removal. Inset: MPIPs on the walls of a vial under magnetic field.

Figure 2 presents the characteristics, including size distribution and mean size, of MIP NPs that were prepared with poly(ethylene-*co*-vinyl alcohol)s, EVALs, that contained various ethylene mole percents. As shown in Figure 2a, the magnetic nanoparticles (MNPs) had diameters of 61 ± 6 nm, while the freshly prepared magnetic peptide imprinted particles (MPIPs; made with 27 mol% EVAL and containing the MNPs) had diameters of 165 ± 28 nm. Interestingly, washing to remove the template actually increased the particle sizes (to 238 ± 30 nm) as measured by dynamic light scattering (DLS), perhaps owing to a swelling of the surface polymer layer. Rebinding of the template led to contraction of the MPIPs to 183 ± 47 nm. Figure 2b shows the mean sizes for particles prepared with different ethylene mole percentages in the EVAL. There is a trend to larger “before washing” sizes with increasing ethylene, but more interestingly, the intermediate ethylene percentages (32 and 38 mol%) gave larger particles after washing (~360 and ~490 nm) and yet the smallest particles on rebinding (~120 and 125 nm). N_2_ adsorption-desorption isotherms (Figure 2c) were used to determine the specific surface area, using the multi-point Brunauer–Emmett–Teller (BET) method. The specific surface areas of MPIPs before and after template removal were 301.4 ± 23.9 m^2^/g and 337.7 ± 35.4 m^2^/g, respectively, for particles prepared using EVAL with ethylene at 27 mol%. The specific surface area in the MPIPs increased slightly upon template removal, as might be expected. The magnetization curves of the MNPs, and MPIPs before and after template removal, plotted in Figure 2d, reveal their superparamagnetic properties; their saturated magnetizations were found to be 58, 48 and 50 emu/g. The inset in Figure 2d shows the accumulation of MPIPs on the side of a vial nearest a magnet. 

Figure S1a in the Supplementary Materials shows the adsorption of peptide on imprinted and non-imprinted nanoparticles made with EVALs with different ethylene contents. EVAL with the lowest ethylene mol% studied, 27 mol%, gave the largest imprinting effectiveness (defined as the ratio of adsorption of template molecules on MMIPs to that on the magnetic non-imprinted polymer composite nanoparticles, MNIPs) in Table S2. Figure S1b plots the isothermal adsorptions of peptide on MPIPs and on MNIPs. The maximum binding on MPIPs and NPIPs was 66.1 ± 12.8 and 39.1 ± 9.4 mg/g, respectively.

Figure S2 displays the viabilities of HepG2 cells under various conditions, but without immunotherapy (i.e. without incubation with NK-92 cells.) Figures S2a and S3 show the viabilities of cells (measured with the CCK-8 kit, normalized to the cell viability obtained with no treatment) incubated for 24 h with four different NPs: unmodified magnetic nanoparticles (MNP), magnetic nanoparticles with surface modified 3-triethoxysilylpropylamine (MNPs/APTES), magnetic nanoparticles with surface APTES and bound merocyanine photosensitizer (MNPs/APTES/MC540), and non-imprinted polymer-coated composite particles (MNIPs). In general, these controls show little effect on cellular viability, though there appears to be a small enhancement with particles containing photosensitizer.

Figure S2b shows HepG2 viability vs irradiation (illumination) duration, for cells in medium containing the free merocyanine photosensitizer MC540. 10 μg/mL MC540 did not suppress the growth of HepG2 cells even under irradiation for as long as 25 min; however, higher concentrations of MC540 dramatically reduced cellular viability when irradiated for longer than 10 min. In contrast, a short duration of irradiation (~5 min) and a large dosage of MC540 actually seemed to promote cell growth after 24 h. Figure S2c plots the viability of HepG2 cells with various concentrations of MEIP/MC540 NPs with or without 15 min of irradiation. The strong phototoxicity of MC540 is retained even when the photosensitizer is incorporated into the peptide-imprinted polymeric nanoparticles. 

**Figure 3:**
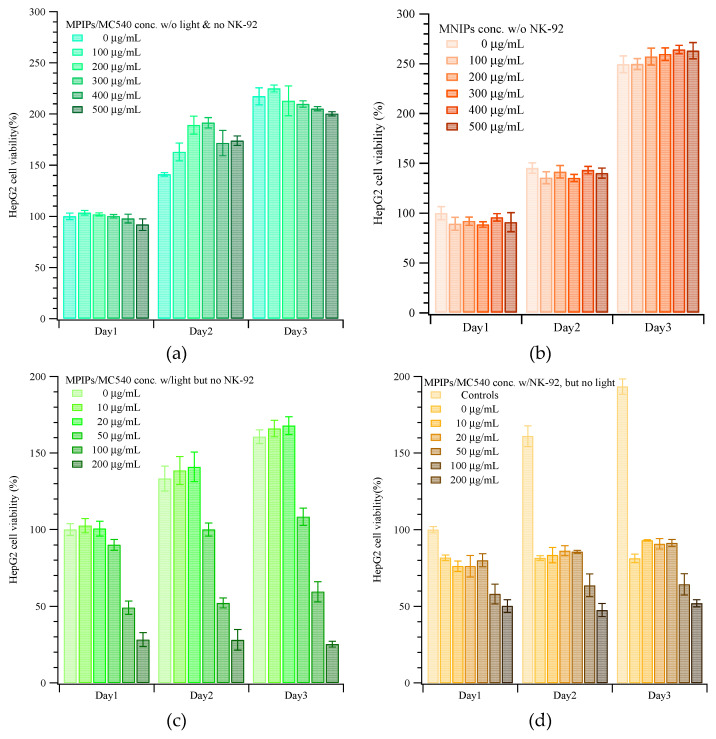

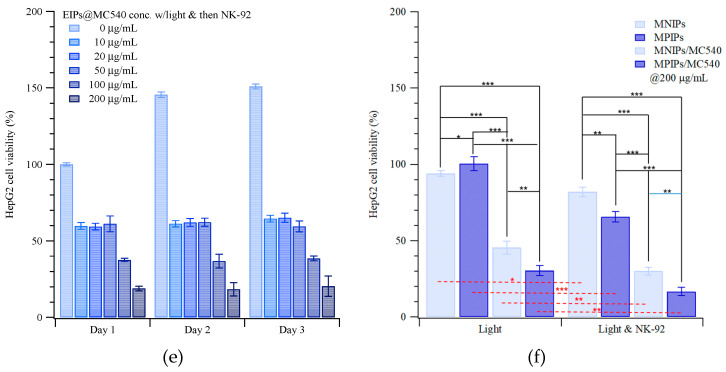
Continuous cellular viability measurements of HepG2 cells incubated with various concentrations of (**a**) MNIPs, (**b**) MPIPs/MC540 without irradiation or NK-92 treatment, (**c**) MPIPs/MC540 with irradiation. (**d**) MPIPs/MC540 with NK-92 treatment, (**e**) MPIPs/MC540 with irradiation or NK-92 treatment, (**f**) Comparison of cellular viability of HepG2 cells with NK-92 cells (2 × 10^4^ cells/well), MNIPs/MC540 or MPIPs/MC540 (200 μg/mL) with or without addition of NK-92 cells NK-92 cells (2 × 10^4^ cells/well) under irradiation (* *p* < 0.05; ** *p* < 0.005; *** *p* < 0.0005).

Figure 3 displays the viabilities of HepG2 cells when dosed with different concentrations of nanoparticles, with or without NK cells and with or without irradiation. Without NK-92 cells and without illumination, the viability of HepG2 cells with various concentrations of MNIPs was about 95%, increasing to 140 and 250% on the second and third days compared to the controls on day 0, as shown in Figure 3a. (Viabilities >100% reflect continued cell growth and division.) In contrast, the viability of HepG2 cells with various concentrations of MPIP/MC540 was about 95%, increasing to 160 and 200% on the second and third days compared to the controls on day 0, as shown in Figure 3b. The phototoxicity of MPIP/MC540 up to 500 μg/mL after 1 day is shown in Figure S2c; the viability for every day administration of a lower concentration of MPIPs/MC540 after 1–3 days is shown in Figure 3c. Treatment with MPIPs/MC540, followed by NK-92 cells after 1 day ((but without irradiation), Figure 3d)), resulted in arrested growth of the cell population, essentially regardless of the MPIPs/MC540 concentration. High MPIPs/MC540 concentrations did result in greater prompt cell death, but even the lowest concentration (zero) showed negligible cell growth. The inhibition of cell growth thus appears to be caused by the NK-92 cells alone [43]. Figure 3e shows the combined effects of phototoxicity and NK-92 treatment. Cells were maintained in media containing different concentrations of MPIP/MC540; each day, cells were treated with photodynamic therapy for 15 min. (The medium was also changed every day, but replaced with fresh medium containing the specified concentration of MPIPs/MC540.) The viabilities are lower than with NK-92 killer cells alone. If MPIPs block PD-L1 and prevent it from binding to PD-1, they should enhance the ability of NK-92 cells to destroy HepG2 cancer cells. Figure 3f demonstrates that a combination of photodynamic therapy and immunotherapy can increase the in vitro suppression of HepG2 cells. 

The viability of HepG2 cells treated with MNIPs with NK-92 cells was reduced by approximately 60%. Furthermore, photodynamic therapy can promote the suppression of HepG2 cells from 60% to about 30%; binding of MPIPs on HepG2 increased suppression to 20%. Similar results can be found in Figure S5 for the treatments of Hepa1-6 cells with MNIPs, MPIPs and MPIPs/MC540 with or without irradiation.

**Figure 4:**
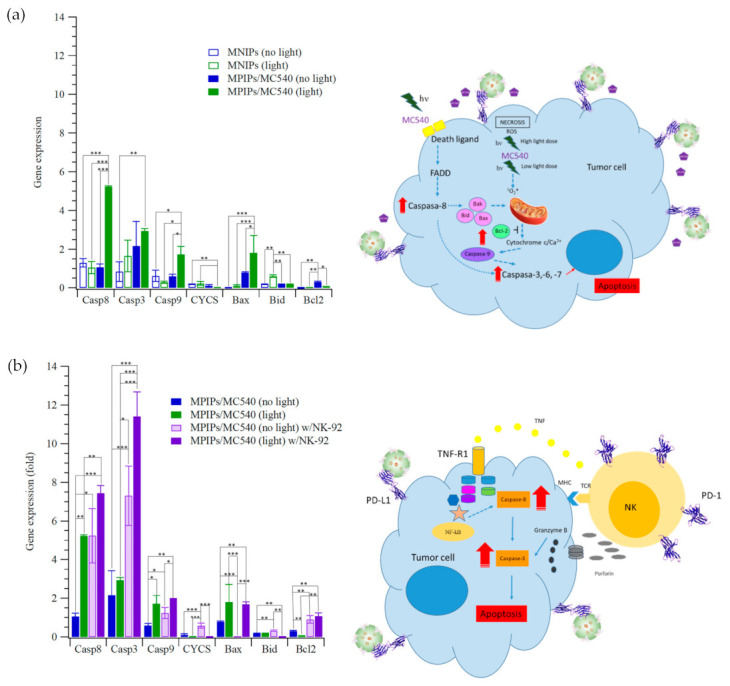
Relative gene expression of cellular apoptosis (Casp8, Casp3, Casp9, CYCS, Bax, Bid and Bcl-2) of HepG2 cells treated with (**a**) MNIPs, MPIPs/MC540 and (**b**) MPIPs/MC540 and additional NK-92 cells (2 × 10^4^ cells/well) under irradiation or not (* *p* < 0.05; ** *p* < 0.005; *** *p* < 0.0005).

The pathway of photodynamic therapy with MPIPs/MC540 was analyzed by qRT-PCR, as presented in Figure 4. This analysis showed that apoptosis was induced using MPIPs/MC540 with irradiation by upregulating caspase-8, caspase-3 caspase-9 and Bax, and downregulation of Bcl-2 protein expression in Figure 4a. Figure 4b shows that gene expressions of caspase-8, caspase-3 caspase-9 and Bax were increased under irradiation of light, compared to no light control group. The apoptosis of HepG2 cells was caused by the activation of Casp9, but not Casp8, possibly indicating the endocytosis of MPIPs/MC540 both with and without the addition of NK-92 cells. ^1^O_2_ was produced inside the HepG2 cells and increased the expression of proapoptotic Bcl family proteins (including Bax and Bid). However, the addition of NK-92 cells for immunotherapy increased the expression antiapoptotic proteins (such as Bcl2), possibly also inhibiting the expression of CYCS and then Casp9. Surprisingly, the combination of immunotherapy and photodynamic therapy dramatically promotes the expression of Casp8 and then Casp3, inducing the apoptosis of HepG2 cells.

**Figure 5:**
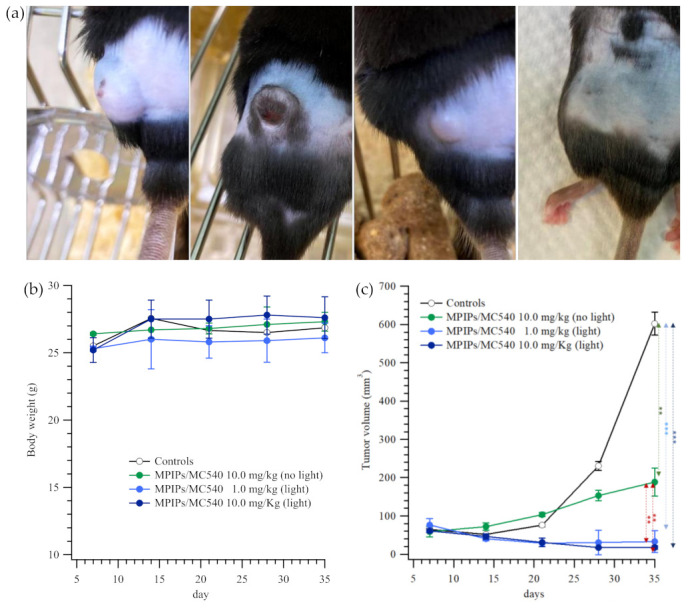
(**a**) Pictures of mice with treatment of PBS, 10.0, 1.0 and 10.0 mg/kg of MPIPs/MC540; the latter two groups were irradiated with 15 min of an LED mini dot light 1 h after injection. (**b**) Body weight of mice during the treatment. (**c**) The tumor size measured on the four groups of mice mentioned in 4 (**a**) (** *p* < 0.005; *** *p* < 0.0005).

The treatment of an animal tumor model using composite nanoparticles is shown in Figure 5. In Figure 5a, photographs of mice treated with PBS (control), 10.0, 1.0 and 10.0 mg/kg of MPIPs/MC540; the latter two groups were irradiated with 15 min of 520 nm light. All mice had initially similar body mass of 24–29 g. The tumor volume for the control increased from 65 to 600 mm^3^ over 11 days. Both the low and high dosages of MPIPs/MC540 accompanied by irradiation with light were effective at decreasing tumor size from 60–75 mm^3^ to 33.1 ± 28.9 and 17.6 ± 4.8 mm^3^, respectively. Surprisingly, the high dosage of MPIPs/MC540 (alone, without irradiation) still suppressed the growth of tumor, keeping it about 1/3 the size of the control. Nonetheless, the most effective treatment combined MPIPs/MC540 with irradiation.

**Figure 6:**
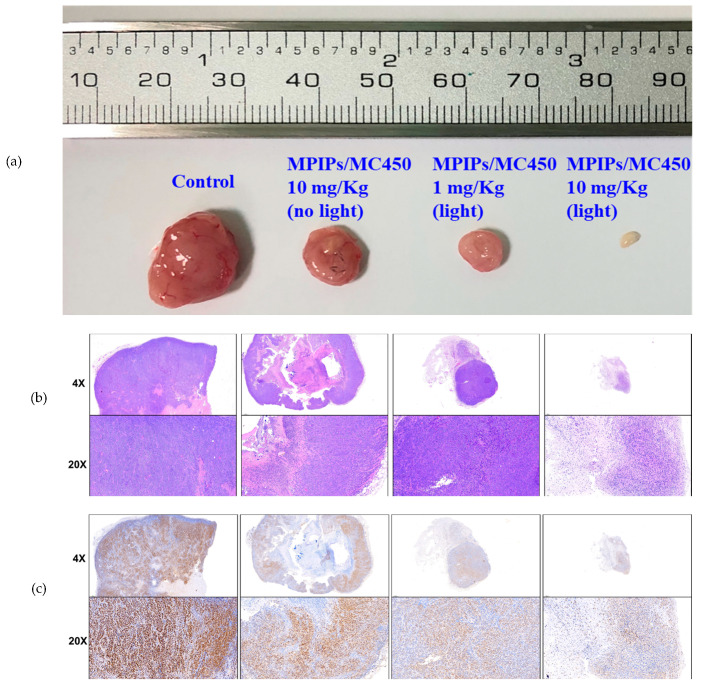
(**a**) Pictures of tumor specimens from the xenograft models with treatment of PBS (control), 10.0, 1.0 and 10.0 mg/kg of MPIPs/MC540; the latter two groups were irradiated with 15 min of an LED mini dot light 1 h after injection. (**b**) Hematoxylin & eosin (HE) staining of above tumor specimens. (**c**) Immunohistochemical (IHC) staining with anti-proliferating cell nuclear antigen (PCNA) primary antibodies.

The morphology and sizes of the tumors are shown in Figure 6, after sacrifice of the mice at 35 days, showing the control tumor, a high dose (10.0 mg/kg) of MPIPs/MC540, a low dose (1.0 mg/kg) with irradiation, and a high dose with irradiation. The tumor sizes are in agreement with the pictures and measurements in Figure 5a,c. The solid tumor had nearly disappeared using high dosages of MPIPs/MC540 combined with irradiation of light. The size of the solid tumor was bigger without irradiation at the same 1.0 mg/kg dose of MPIPs/MC540.

Hematoxylin and eosin (HE) stains and immunohistochemistry (IHC) stains (using proliferating cell nuclear antigen (PCNA) antibody) have been used for recognizing the growth and proliferation of cancer cells, respectively. The results of HE stains were shown for control, low/high dosages of MPIPs/MC540 with irradiation of light and low dosages of MPIPs/MC540 without irradiation in Figure 6b. Nuclei and cytoplasm are purple and pink, respectively, using HE stains for live cancer tissue. Comparing control and low/high dosages of MPIPs/MC540 with/without irradiation of light, the purple distribution was looser at high dosages of MPIPs/MC540 with irradiation of light. This supports that, at high dosages of MPIPs/MC540 with irradiation of light, tumor cell growth is suppressed. PCNA immunohistochemistry with antigen retrieval was used to measure the proliferation of cells. In Figure 6c, the brown and blue-purple were PCNA and hematoxylin stains, respectively; tumor proliferation was slightly reduced without irradiation of light at high dosages of MPIPs/MC540, but more effectively reduced under irradiation of light at low and high dosages of MPIPs/MC540.

## 3. Materials and Methods

### 3.1. Reagents and Chemicals

One peptide of PD-L1 (EDLKVQHSSYRQRA) was ordered from Yao-Hong Biotechnology Inc. (HPLC grade, New Taipei City, Taiwan). Poly(ethylene-*co*-vinyl alcohol), EVAL, with ethylene 27, 32, 38 and 44 mol%, 3-triethoxysilylpropylamine (APTES), merocyanine 540 (MC540) and RT-PCR primers, which were listed in Table S1, were from Sigma-Aldrich Co. (St. Louis, MO, USA). Iron (III) chloride 6-hydrate (97%), iron(II) sulphate 7-hydrate (99.0%) and dimethyl sulfoxide (DMSO) were from Panreac (Barcelona, Spain). DMSO was used as the solvent to dissolve EVAL polymer particles in the concentration of 1 wt%. Absolute ethyl alcohol was from J.T. Baker (ACS grade, Phillipsburg, NJ, USA). Both human hepatoblastoma (HepG2, #60364) and human natural killer cell (NK-92, #60414) were obtained from Bioresource Collection and Research Centre (BCRC), Taiwan.

### 3.2. Formation of Multifunctional Magnetic Peptide-Imprinted Composite Nanoparticles

The synthesis of multifunctional magnetic peptide-imprinted composite nanoparticles included the following steps: (1) magnetic nanoparticles, synthesized by co-precipitation of a mixture of iron (III) chloride 6-hydrate and iron (II) sulfate 7-hydrate by sodium hydroxide, were repeatedly washed while adsorbed on a magnetic plate [13]. (2) Two g of magnetic nanoparticles were mixed with 90 mL of ethanol to form a uniformly dispersed solution, and 180 μL APTES was then added. The mixture was stirred in a water bath at 90 °C for 90 min. Then, the APTES/MNPs were washed with 10 mL 95% alcohol for 5 min (3×), separated with a magnet after each washing. Three mL of MC540 (1.0 mg/mL) and 10 g of APTES/MNPs were mixed for 10 min and then washed with deionized water twice. (3) Peptide was dissolved in DMSO, at concentrations of 0.2, 2, 20, 100 and 200 μg/mL. 250 μL EVAL/DMSO solution was added into the same volume of peptide solution to form a clear EVAL solution, and 10 mg of the composite nanoparticles were then added. The EVAL was precipitated by dispersing 0.5 mL EVAL solution into 10 mL deionized water; then template was removed by washing in 10 mL deionized water 15 min (3×), separating the multifunctional magnetic peptide-imprinted polymer composite nanoparticles (MPIPs/MC540) magnetically after each washing. The magnetic non-imprinted polymer composite nanoparticles (MNIPs) were prepared identically, but without peptide addition.

### 3.3. Cytotoxicity Test of HepG2 Cells with Magnetic Peptide-Imprinted Composite Nanoparticles and NK-92 Cells

#### 3.3.1. MTT Assay for Cell Viability

HepG2 cells were cultured in 90% of a 1:1 ratio mixture of Dulbecco’s modified Eagle’s medium (DMEM) and Ham’s F12 medium with 10% heat-inactivated fetal bovine serum (FBS), supplemented with 0.4 mg/mL G418 (Geneticin) at 37 °C and 5% CO_2_. For the cytotoxicity experiments 10 μL of 7.5.0x10^4^ HepG2 cells and 190 μL culture medium per well (7.5 × 10^3^ HepG2 cells per well) were seeded in 96 well culture plates and then incubated at 37 °C in 5% CO_2_ for 24 h. Various concentrations of nanoparticles were added to each well at 37 °C for 24 h. 20 μL MTT (3-(4,5-dimethylthiazol-2-yl)-2,5 diphenyltetrazolium bromide, a yellow tetrazole) solution in phosphate buffered saline (PBS) was added to each well after 24 h, and then incubated in 5% CO_2_ for 3 h at 37 °C. The solution was removed from each well. Then, 100 μL dimethyl sulfoxide (DMSO) was added to each well and incubated at 37 °C for 30 min in the dark, until cells have lysed and purple crystals have dissolved. The absorption intensities were measured by an ELISA reader (CLARIOstar, BMG Labtech, Offenburg, Germany) at 450 nm (I_450_), and the reference absorption (I_ref_, to account for turbidity and scattering) was obtained at 650 nm. The cellular viability (%) was then calculated from the ratio of effective absorption (I_450_–I_ref_) to controls.

#### 3.3.2. Photodynamic Therapy (PDT)

The HepG2 cells at a density of 5 × 10^4^ cells/mL were added to a 24-well culture plate and incubated for 24 h at 37 °C in 5% CO_2_. Various concentrations of MNPs, APTES/MNPs, MNPs/MC540, and MNIP/MC540, MPIPs/MC540 nanoparticles were added to HepG2 cells, respectively. The well plate was exposed to photodynamic light irradiation using an LED mini dot light (HO HUA Electronic Components Company, Kaohsiung, Taiwan) for 15 min, with wavelengths from 460 to 580 nm and peak intensity at 520 nm. The distance was 1.5 cm between the LED device (50 W/m^2^) and the cell plate. Then, the well culture plate was incubated for 24 h at 37 °C in 5% CO_2_. The cellular viability of HepG2 cells was analyzed using MTT assay (see Section 3.3.1).

#### 3.3.3. Immunotherapy with NK-92 Cells

NK-92 cells were cultured in RPMI1640 with l-glutamine and 10% FBS at 37 °C and 5% CO_2_. After PDT, a CCK-8 cell counting kit (Sigma Aldrich, Kumamoto, Japan) was used to repeatedly assess cell viability. Particles were removed from wells and 500 μL of CCK-8 solution was added before the absorption intensity measurements by an ELISA reader, as described in Section 3.3.1. Only NK-92 (2 × 10^4^ cells/well) were added on the second and third day.

### 3.4. Gene Expression of HepG2 Cells Treated with MPIPs, NK92 and PDT

The sequence (5’–3’) of primers for GAPDH, Caspase 8, Caspase 3, Caspase 9, Cytochrome C (CYCS), Bax, Bid, Bcl-2 was listed in Table S1. The total RNA extraction from the HepG2 cells cultured one day after UV irradiation was performed using the KingFisher Total RNA Kit and the KingFisher mL magnetic particle processors, both from Thermo Scientific (Vantaa, Finland). Complementary DNA was obtained following a Deoxy+ real-time 2× SYBR green RT-PCR kit (Yeastern Biotech Co., Ltd., Taipei, Taiwan) protocol. The RT-PCR was then performed in a PikoReal RT-PCR system (Thermo Scientific). Relative gene expression was determined using the ΔΔCq method [44] and normalized to a reference gene (GAPDH) and to a control (HepG2).

### 3.5. Animal Model and Immunohistochemical Staining

The animal experiments were approved by the Institutional Animal Care and Use Committees (IACUC) of National University of Kaohsiung (NUK) (protocol No. 10708, 10 November 2018) and performed in accordance with the Association for Assessment and Accreditation of Laboratory Animal Care guidelines (http://www.aaalac.org, accessed on 25 May 2021). To generate murine subcutaneous tumors, (1 × 10^6^ cells/mouse) Hepa1-6 tumor cells were transplanted into the right flank of C57CL/6 mice. When the tumor volumes were around 50∼100 mm^3^, the mice were randomly divided into four groups and were intravenously injected with PBS, MEIPs/MC540 (1.0 or 10.0 mg/kg). One hour after injection, the low (1.0 mg/kg) and (10.0 mg/kg) groups were then irradiated with light (wavelength 520 nm, intensity 1W) for 15 min. This treatment was performed twice a week for 5 weeks. On fifth week after the start of treatment, tumors were removed. Tumors were measured twice weekly, and the tumor volume (mm^3^) was calculated as (long diameter × short diameter^2^)/2. Once the mice exhibit signs of impaired health or when the volume of the tumor exceeded 1.5 cm^3^, the mice were euthanized with CO_2_.

Tumor specimens from the xenograft models were cut into 5 μm slices, fixed in 10% neutral buffered formalin, and embedded in paraffin. Slides were preincubated in 5% goat serum (Abcam, Cambridge, UK) in PBS and immunostained with anti-proliferating cell nuclear antigen (PCNA; GeneTex, Irvine, CA, USA) primary antibodies (both diluted 1:500 at 37 °C for 2 h. Slides were treated with hematoxylin for 30 s for visualization under a light microscope (UltraVision System; Thermo).

### 3.6. Data Analysis

All experiments were carried out in triplicate and data are expressed as means ± standard deviation. The cellular viability, gene expression data and tumour volume were analyzed with Student’s t-test. Statistical significance was set at a *p*-value of less 0.05, significant as *p* < 0.05, highly significant as *p* < 0.005, extremely significant as *p* < 0.0005.

## 4. Conclusions

In this work, molecularly imprinted polymer (MIP) particles were synthesized to target the programmed death receptor (PD-L1) on HepG2 cells, and a photosensitizer (MC540) was incorporated to enhance the efficacy of photodynamic therapy. Additionally, blocking the PD-L1 protein with the MIP particles increased the functionality of natural killer (NK-92) cells. A biomolecular pathway investigation of HepG2 cells revealed that the singlet oxygen produced by irradiation promoted the expression of proapoptotic Bcl family proteins. Furthermore, the NK-92 cells further promoted the expression of Casp8 in the apoptosis of HepG2 cells. To summarize, this work demonstrated the efficacy of this combined therapy on a tumor model in mice and identified pathways and mechanisms of action.

## Data Availability

The authors confirm that the data supporting the findings of this study are available within the article and its supplementary materials.

## Supplementary Materials

The following are available online at https://www.mdpi.com/article/10.3390/ph14060508/s1, Experimental Section S1.1 Characterization of magnetic peptide-imprinted polymer composite nanoparticles; Table S1: The sequence of primers used in this work, including GAPDH, Casp8, Casp3, Casp9, CYCS, Bax, Bid, and Bcl-2; Table S2: Prescreening of the binding of peptide molecules to magnetic peptide- and non-imprinted poly(ethylene-*co*-vinyl alcohol) nanoparticles; Figure S1. (a) Adsorption of peptide (50 μg/mL) on the MPIPs with imprinted onto various ethylene mol% of EVAL (b) Isothermal adsorption of MPIPs and MNIPs with various of peptide concentrations; Figure S2. Cellular viability of HepG2 cells with (a) various concentrations of MNPs, MNP/APTES, MNP/APTES/MC540, and MNIPs/MC540; (b) various concentrations of free MC540 and irradiation durations; (c) various concentrations of MPIPs/MC540 with and without irradiation; Figure S3. Optical, DAPI stained and merged images (from left to right columns) of (a) HepG2 cells; with MNPs (b); with MNPs/APTES (c), with MNPs/APTES/MC540 (d); and MNIPs/MC540 (e); Figure S4. (a) Immunostaining of CD-133 on HepG2 cells, (b) MPIPs/MC540 and (c) their merge image; Figure S5. Continuous cellular viability measurements of Hepa1-6 cells incubated with 100 μg/mL of MNIPs, MPIPs and MPIPs/MC540 (a) without or (b) with irradiation.

## References

[1] Lee M.-H., Thomas J.L., Wang H.-Y., Chang C.-C., Lin C.-C., Lin H.-Y. (2012). Extraction of resveratrol from polygonum cuspidatum with magnetic orcinol-imprinted poly(ethylene-co-vinyl alcohol) composite particles and their in vitro suppression of human osteogenic sarcoma (HOS) cell line. J. Mater. Chem..

[2] Lee M.-H., Thomas J.L., Chen Y.-C., Wang H.-Y., Lin H.-Y. (2012). Hydrolysis of Magnetic Amylase-Imprinted Poly(ethylene-co-vinyl alcohol) Composite Nanoparticles. ACS Appl. Mater. Interfaces.

[3] Lin H.-Y., Ho M.-S., Lee M.-H. (2009). Instant formation of molecularly imprinted poly(ethylene-co-vinyl alcohol)/quantum dot composite nanoparticles and their use in one-pot urinalysis. Biosens. Bioelectron..

[4] Huang C.-Y., Tsai T.-C., Thomas J.L., Lee M.-H., Liu B.-D., Lin H.-Y. (2009). Urinalysis with molecularly imprinted poly(ethylene-co-vinyl alcohol) potentiostat sensors. Biosens. Bioelectron..

[5] Lee M.H., Thomas J.L., Su Z.L., Yeh W.K., Monzel A.S., Bolognin S., Schwamborn J.C., Yang C.H., Lin H.Y. (2020). Epitope imprinting of alpha-synuclein for sensing in Parkinson’s brain organoid culture medium. Biosens Bioelectron..

[6] Lee M.-H., Lin C.-C., Thomas J.L., Li J.-A., Lin H.-Y. (2021). Cellular reprogramming with multigene activation by the delivery of CRISPR/dCas9 ribonucleoproteins via magnetic peptide-imprinted chitosan nanoparticles. Mater. Today Bio.

[7] Lee M.-H., Lin C.-C., Thomas J.L., Chan C.-K., Lin H.-Y. (2021). Epitope recognition of magnetic peptide-imprinted chitosan composite nanoparticles for the extraction of CRISPR/dCas9a proteins from transfected cells. Nanotechnology.

[8] Lee M.-H., Thomas J.L., Chang Y.-C., Tsai Y.-S., Liu B.-D., Lin H.-Y. (2016). Electrochemical sensing of nuclear matrix protein 22 in urine with molecularly imprinted poly(ethylene-co-vinyl alcohol) coated zinc oxide nanorod arrays for clinical studies of bladder cancer diagnosis. Biosens. Bioelectron..

[9] Chen W.-J., Lee M.-H., Thomas J.L., Lu P.-H., Li M.-H., Lin H.-Y. (2013). Microcontact Imprinting of Algae on Poly (ethylene-co-vinyl alcohol) for Biofuel Cells. ACS Appl. Mater. Interfaces.

[10] Lee M.-H., Thomas J.L., Chen W.-J., Li M.-H., Shih C.-P., Lin H.-Y. (2015). Fabrication of bacteria-imprinted polymer coated electrodes for microbial fuel cells. ACS Sustain. Chem. Eng..

[11] Lin H.-Y., Hsu C.-Y., Thomas J.L., Wang S.-E., Chen H.-C., Chou T.-C. (2006). The microcontact imprinting of proteins: The effect of cross-linking monomers for lysozyme, ribonuclease A and myoglobin. Biosens. Bioelectron..

[12] Lee M.-H., Liu K.-T., Thomas J.L., Su Z.-L., O’Hare D., van Wuellen T., Chamarro J.M., Bolognin S., Luo S.-C., Schwamborn J.C. (2020). Peptide-Imprinted Poly(hydroxymethyl 3,4-ethylenedioxythiophene) Nanotubes for Detection of α Synuclein in Human Brain Organoids. ACS Appl. Nano Mater..

[13] Lee M.-H., Thomas J.L., Liao C.-L., Jurcevic S., Crnogorac-Jurcevic T., Lin H.-Y. (2018). Epitope recognition of peptide-imprinted polymers for Regenerating protein 1 (REG1). Sep. Purif. Technol..

[14] Refaat D., Aggour M.G., Farghali A.A., Mahajan R., Wiklander J.G., Nicholls I.A., Piletsky S.A. (2019). Strategies for molecular imprinting and the evolution of MIP nanoparticles as plastic antibodies—Synthesis and applications. Int. J. Mol. Sci..

[15] Nishino H., Huang C.S., Shea K.J. (2006). Selective protein capture by epitope imprinting. Angew. Chem. Int. Ed..

[16] Zeng Z., Hoshino Y., Rodriguez A., Yoo H., Shea K.J. (2009). Synthetic polymer nanoparticles with antibody-like affinity for a hydrophilic peptide. ACS Nano.

[17] Zhao X.-L., Li D.-Y., He X.-W., Li W.-Y., Zhang Y.-K. (2014). An epitope imprinting method on the surface of magnetic nanoparticles for specific recognition of bovine serum album. J. Mater. Chem. B.

[18] Qin Y.-P., Jia C., He X.-W., Li W.-Y., Zhang Y.-K. (2018). Thermosensitive Metal Chelation Dual-Template Epitope Imprinting Polymer Using Distillation–Precipitation Polymerization for Simultaneous Recognition of Human Serum Albumin and Transferrin. ACS Appl. Mater. Interfaces.

[19] Bossi A.M., Sharma P.S., Montana L., Zoccatelli G., Laub O., Levi R. (2012). Fingerprint-Imprinted Polymer: Rational Selection of Peptide Epitope Templates for the Determination of Proteins by Molecularly Imprinted Polymers. Anal. Chem..

[20] Iskierko Z., Sharma P.S., Noworyta K.R., Borowicz P., Cieplak M., Kutner W., Bossi A.M. (2019). Selective PQQPFPQQ Gluten Epitope Chemical Sensor with a Molecularly Imprinted Polymer Recognition Unit and an Extended-Gate Field-Effect Transistor Transduction Unit. Anal. Chem..

[21] Lach P., Cieplak M., Majewska M., Noworyta K.R., Sharma P.S., Kutner W. (2019). “Gate Effect” in p-Synephrine Electrochemical Sensing with a Molecularly Imprinted Polymer and Redox Probes. Anal. Chem..

[22] Lee M.-H., Thomas J.L., Liao C.-L., Jurcevic S., Crnogorac-Jurcevic T., Lin H.-Y. (2017). Polymers imprinted with three REG1B peptides for electrochemical determination of Regenerating Protein 1B, a urinary biomarker for pancreatic ductal adenocarcinoma. Microchim. Acta.

[23] Pan J., Chen W., Ma Y., Pan G. (2018). Molecularly imprinted polymers as receptor mimics for selective cell recognition. Chem. Soc. Rev..

[24] Piletsky S., Canfarotta F., Poma A., Bossi A.M., Piletsky S. (2020). Molecularly Imprinted Polymers for Cell Recognition. Trends Biotechnol..

[25] Guan J., Lim K.S., Mekhail T., Chang C.-C. (2017). Programmed death ligand-1 (PD-L1) expression in the programmed death receptor-1 (PD-1)/PD-L1 blockade: A key player against various cancers. Arch. Pathol. Lab. Med..

[26] Lau J., Cheung J., Navarro A., Lianoglou S., Haley B., Totpal K., Sanders L., Koeppen H., Caplazi P., McBride J. (2017). Tumour and host cell PD-L1 is required to mediate suppression of anti-tumour immunity in mice. Nat. Commun..

[27] Zou W., Wolchok J.D., Chen L. (2016). PD-L1 (B7-H1) and PD-1 pathway blockade for cancer therapy: Mechanisms, response biomarkers, and combinations. Sci. Transl. Med..

[28] Milling L., Zhang Y., Irvine D.J. (2017). Delivering safer immunotherapies for cancer. Adv. Drug Deliv. Rev..

[29] Jo S.D., Nam G.-H., Kwak G., Yang Y., Kwon I.C. (2017). Harnessing designed nanoparticles: Current strategies and future perspectives in cancer immunotherapy. Nano Today.

[30] Qian H., Liu B., Jiang X. (2018). Application of nanomaterials in cancer immunotherapy. Mater. Today Chem..

[31] Duan X., Chan C., Lin W. (2019). Nanoparticle-Mediated Immunogenic Cell Death Enables and Potentiates Cancer Immunotherapy. Angew. Chem. Int. Ed..

[32] Liu Q., Tian J., Tian Y., Sun Q., Sun D., Wang F., Xu H., Ying G., Wang J., Yetisen A.K. (2021). Near-Infrared-II Nanoparticles for Cancer Imaging of Immune Checkpoint Programmed Death-Ligand 1 and Photodynamic/Immune Therapy. ACS Nano.

[33] Robertson C.A., Evans D.H., Abrahamse H. (2009). Photodynamic therapy (PDT): A short review on cellular mechanisms and cancer research applications for PDT. J. Photochem. Photobiol. B Biol..

[34] Hoebeke M., Piette J., van de Vorst A. (1991). Photosensitized production of singlet oxygen by merocyanine 540 bound to liposomes. J. Photochem. Photobiol. B Biol..

[35] Firczuk M., Nowis D., Gołąb J. (2011). PDT-induced inflammatory and host responses. Photochem. Photobiol. Sci..

[36] Wang H., Liu Z., Wang S., Dong C., Gong X., Zhao P., Chang J. (2014). MC540 and upconverting nanocrystal coloaded polymeric liposome for near-infrared light-triggered photodynamic therapy and cell fluorescent imaging. ACS Appl. Mater. Interfaces.

[37] Idris N.M., Gnanasammandhan M.K., Zhang J., Ho P.C., Mahendran R., Zhang Y. (2012). In vivo photodynamic therapy using upconversion nanoparticles as remote-controlled nanotransducers. Nat. Med..

[38] Xu J., Yang P., Sun M., Bi H., Liu B., Yang D., Gai S., He F., Lin J. (2017). Highly emissive dye-sensitized upconversion nanostructure for dual-photosensitizer photodynamic therapy and bioimaging. ACS Nano.

[39] Trinchet J.-C., Beaugrand M. (1997). Treatment of hepatocellular carcinoma in patients with cirrhosis. J. Hepatol..

[40] Singh A.K., Kumar R., Pandey A.K. (2018). Hepatocellular carcinoma: Causes, mechanism of progression and biomarkers. Curr. Chem. Genom. Transl. Med..

[41] Bruix J., Reig M., Sherman M. (2016). Evidence-based diagnosis, staging, and treatment of patients with hepatocellular carcinoma. Gastroenterology.

[42] Heimbach J.K., Kulik L.M., Finn R.S., Sirlin C.B., Abecassis M.M., Roberts L.R., Zhu A.X., Murad M.H., Marrero J.A. (2018). AASLD guidelines for the treatment of hepatocellular carcinoma. Hepatology.

[43] Lee M.-H., Liu K.-H., Thomas J.L., Chen J.-R., Lin H.-Y. (2019). Immunotherapy of Hepatocellular Carcinoma with Magnetic PD-1 Peptide-Imprinted Polymer Nanocomposite and Natural Killer Cells. Biomolecules.

[44] Pfaffl M.W. (2001). A new mathematical model for relative quantification in real-time RT-PCR. Nucleic Acids Res..

