# Supplementary material for: Synthesis of Multifunctional Nanoparticles for the Combination of Photodynamic Therapy and Immunotherapy
Source: Pharmaceuticals (Basel). 2021 May 26;14(6):508. doi: 10.3390/ph14060508 (PMC8228393; doi:10.3390/ph14060508)
Supplement: Supplementary file 1 [file pharmaceuticals-14-00508-s001.zip › pharmaceuticals-1223950-supplementary.pdf]

pharmaceuticals

MDPI

Supporting Information:

Synthesis of Multifunctional Nanoparticles for the Combination of Photodynamic Therapy and Immunotherapy

Mei-Hwa Lee[1,*], James L. Thomas[2], Jin-An Li[3], Jyun-Ren Chen[3], Tzong-Liu Wang[3] and Hung-Yin Lin[3,*]

[1]Department of Materials Science and Engineering, I-Shou University, Kaohsiung 84001, Taiwan
[2]Department of Physics and Astronomy, University of New Mexico, Albuquerque, NM 87131, USA
[3]Department of Chemical and Materials Engineering, National University of Kaohsiung, Kaohsiung 81148, Taiwan

S1. Experimental Section

S1.1 Characterization of magnetic peptide-imprinted polymer composite nanoparticles

Magnetic, peptide- and non-imprinted EVAL composite particles were examined with a particle sizer (90Plus, Brookhaven Instruments Co., NY). The measurement of the particle size distribution was based on dynamic light scattering (DLS) at 25°C with 3 min duration data collection at the 90° detection angle. The average count rate of the background was 15 kcps and that of each measurement was between 20 ~ 500 kcps. The magnetization of magnetic, peptide-imprinted polymeric nanoparticles before and after template removal was measured with a magnetic property measurement MPMS XL-7 system (Quantum Design, San Diego, CA) at 298 K in ±15000 Gauss. The adsorption of peptide on nanoparticles was examined with a high-pressure liquid chromatography (HPLC) system. Peptide solution (1.0 mg/mL) in water was diluted to various concentrations for calibration. The solutions were passed through a 0.22 m PVDF syringe filter (Advangene Consumables Inc., USA) and then injected into a high-pressure liquid chromatography (HPLC) system for analysis of peptide. The separation was performed on a Kromasil 100-5 C18 column (5 μm particle size, pore size 100 Å, 25 cm x 4.6 mm I.D., SigmaAldrich, USA). The sample (20 L) was eluted with a mobile phase composed of 0.1% TFA in 100% water (solvent A) and 0.1% TFA in 100% acetonitrile (solvent B). The gradient profile for peptide was: 0-0.1 min, 67% A, 33% B; 0.1-25 min, 42% A, 58% B; 25.01-30 min, B 100%. The flow rate and detection wavelength were set to be 1.0 mL/min and 220 nm, respectively, and the retention times for the peptide was 4.49 min.

Table S1. The sequence of primers used in this work, incluidng GAPDH, Casp8, Casp3, Casp9, CYCS , Bax, Bid, and Bcl-2.

Primer	Sequence	
	Forward	Reverse
GAPDH	ACAGTTGCCATGTAGACC	TTTTTGGTTGAGCACAGG
Casp8	CTACAGGGTCATGCTCTATC	ATTTGGAGATTTCCTCTTGC
Casp3	AAAGCACTGGAATGACATC	CGCATCAATTCCACAATTTC
Casp9	CTCTACTTTCCCAGGTTTTG	TTTCACCGAAACAGCATTAG
CYCS	AAGAACAAAGGCATCATCTG	GCTATTAAGTCTGCCCTTTC
Bax	AACTGGACAGTAACATGGAG	TTGCTGGCAAAGTAGAAAAG
Bid	CTTAGCCAGAAATGGGATG	AGTCACAGCTATCTTCCAG
Bcl-2	GATTGTGGCCTTCCTTTGAG	GTTCCACAAAGGCATCC

Table S2. Prescreening of the binding of peptide molecules to magnetic peptide- and non-imprinted poly(ethylene-*co*-vinyl alcohol) nanoparticles. Imprinting effectiveness for four different ethylene mol-% are shown. Standard deviations are based on three individual measurements.

EVAL (ethylene mole %)	Peptide adsorption (g/g)		
	MPIPs	MNIPs	IF
**27**	**27.71± 1.97**	**13.88± 6.20**	**2.00**
32	38.33± 0.88	42.17± 0.57	0.91
38	59.35± 0.24	58.05± 2.54	1.02
44	58.78±0.83	56.86± 0.31	1.03

MPIPs: Magnetic peptide-imprinting polymer nanoparticles; MNIPs: Magnetic non-imprinting polymers. nanoparticles; IF: Imprinting effectiveness. ˙

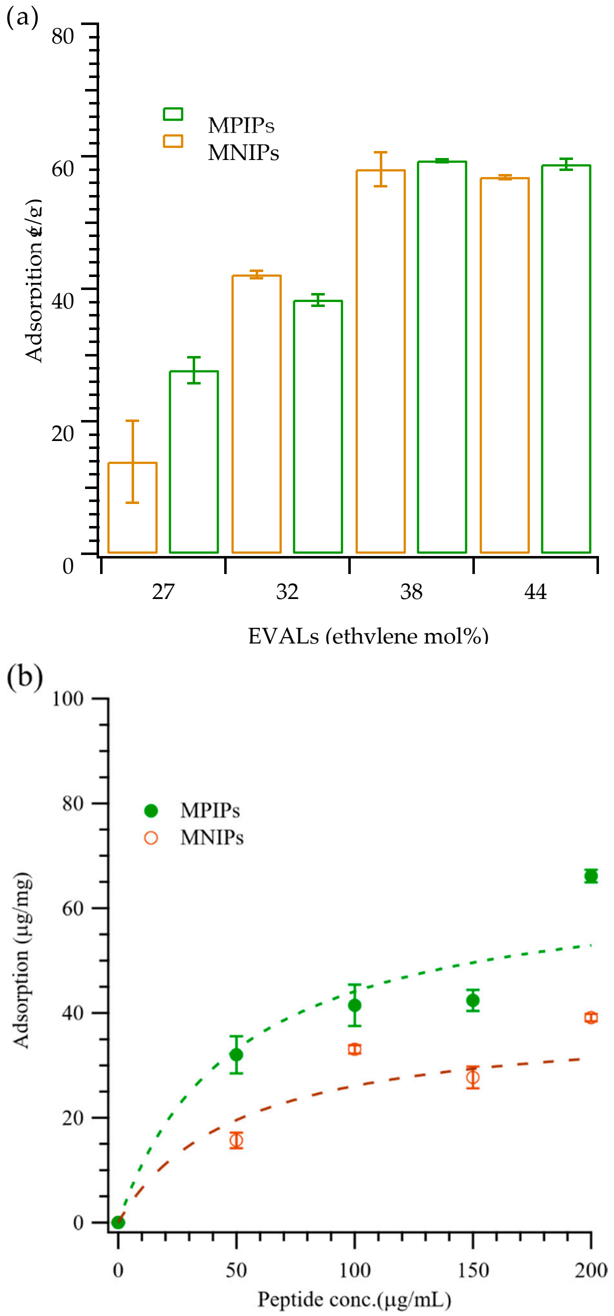

Figure S1. (**a**) Adsorption of peptide (50 g/mL) on the MPIPs with imprinted onto various ethylene mol% of EVAL (**b**) Isothermal adsorption of MPIPs and MNIPs with various of peptide concentrations.

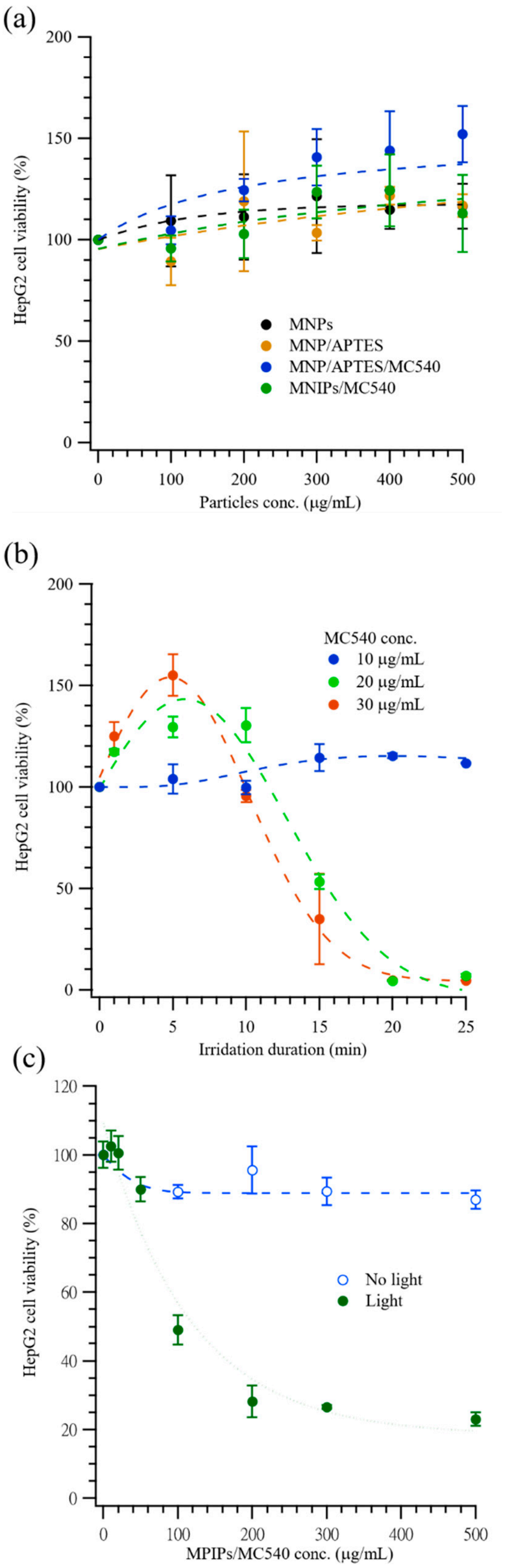

Figure S2. Cellular viability of HepG2 cells with (**a**) various concentrations of MNPs, MNP/APTES, MNP/APTES/MC540, and MNIPs/MC540; (**b**) various concentrations of free MC540 and irradiation durations; (**c**) various concentrations of MPIPs/MC540 with and without irradiation. In all figures, error bars represent the standard deviation of 3 or more wells.

.

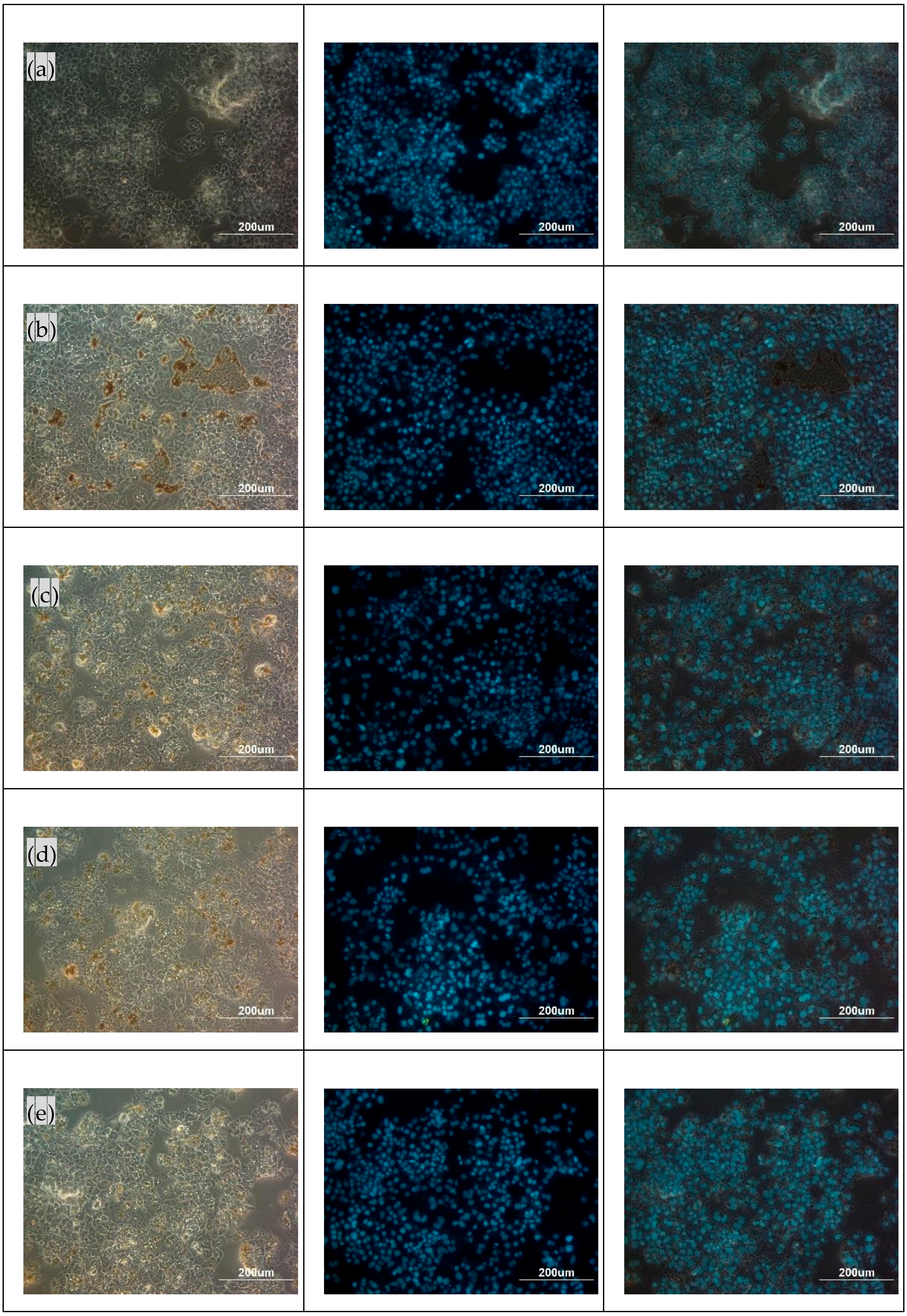

Figure S3. Optical, DAPI stained and merged images (from left to right columns) of (**a**) HepG2 cells; with MNPs (**b**); with MNPs/APTES (**c**), with MNPs/APTES/MC540 (**d**); and MNIPs/MC540 (**e**).

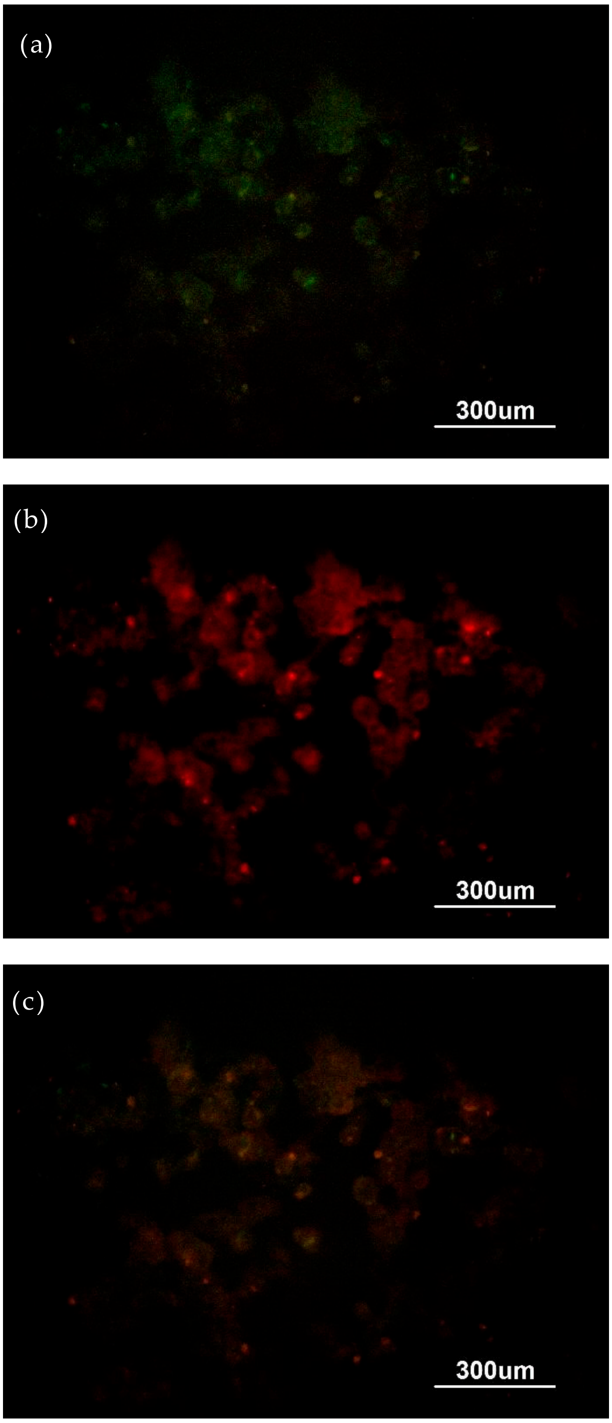

Figure S4. (a) Immunostaining of CD-133 on HepG2 cells, (b) MPIPs/MC540 and (c) their merge image. .

(a)

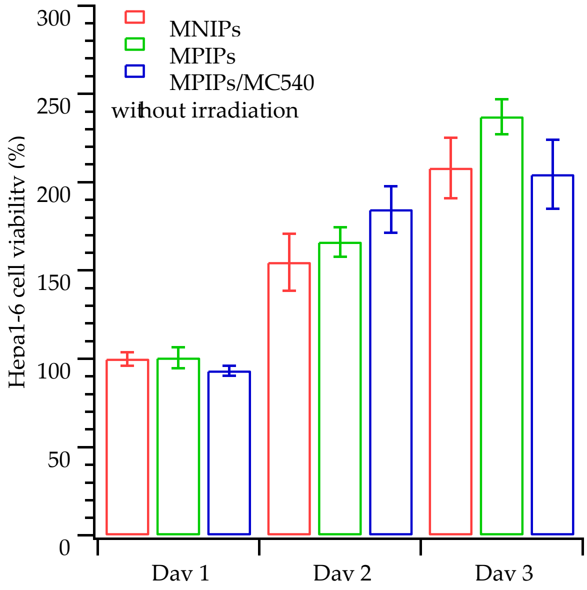

(b)

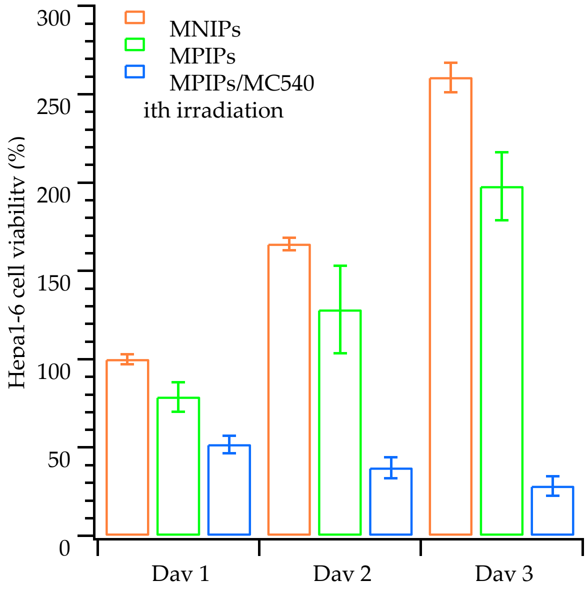

Figure S5. Continuous cellular viability measurements of Hepa1-6 cells incubated with 100 g/mL of MNIPs, MPIPs and MPIPs/MC540 (a) without or (b) with irradiation.
